# Effects of cultivation practice on floristic and flowering diversity of spontaneously growing plant species on arable fields

**DOI:** 10.1002/ece3.8223

**Published:** 2021-10-13

**Authors:** Jörg Hoffmann, Tim Wahrenberg

**Affiliations:** ^1^ Julius Kühn‐Institut Federal Research Centre for Cultivated Plants JKI Institute for Strategies and Technology Assessment Kleinmachnow Germany

**Keywords:** arable fields, conventional, floristic diversity, flowering diversity, organic, smallholder farmland

## Abstract

In the past, the floristic diversity of arable fields has been described in terms of species diversity (SD) and their degree of coverage (C), but never in combination with the recording of the actually flowered species (FS) and their flowering intensity (FI) to striking differences in the cultivation methods on arable land. In relation to SD and C, however, FS and FI may provide important additional information on the functional biodiversity of fields. The aim was therefore to investigate the effects of (a) conventional, (b) organic, and (c) smallholder (never application of herbicides) on the floristic diversity. Using a region in Germany, we investigated SD, C, FS, and FI synchronously in (a), (b), and (c), by 356 vegetation surveys (5 × 5 m plots) conducted in spring and summer in 2019 in winter cereals. Statistical tests were used to analyze the differences between (a), (b), and (c). The medians were used to compare the floristic diversity of (a), (b), and (c) and finally relationships of FS and FI to SD were analyzed in relation to the cultivation methods. Significant differences in SD, C, FS, and FI were found between the (a), (b), and (c) in spring and summer characterized by sharp declines from (c) to (b) to (a). A drastic reduction in floristic diversity from (c) 100 to (b) 52 to (a) 3 was determined. Plants in flower (FS, FI) were very poorly in (a), moderately well to well in (b), and well to very well represented in (c). (C) to (a) was characterized by a sharp decline and from (a) to (b) by sharp increase in floristic diversity. With current acreage proportions of (a) in mind, this would affect, about one third of land area in Germany, associated with a drastic reduction in functional biodiversity for insects.

## Graphical abstract

Significant differences in species diversity (SD), coverage (C), flowering species (FS) and flowering intensity (FI) were found between the three fundamentally different cultivation methods of arable fields: (a) conventional farmland; (b) organic farmland; and (c) smallholder farmland that had never been treated with pesticides. These were characterised by sharp declines in SD, C, FS and FI from (c) to (b) to (a), summarised in a relation of the floristic diversity from 100 to 52 to 3 associated with a drastic reduction in functional biodiversity for insects.

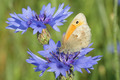

## 1 INTRODUCTION

Agricultural land—the main habitat type—accounts for the largest share of the world's land area (38%); 10.9% of the entire land area is arable land (Weijden et al., 2010). A quarter of the land in the EU is used for agriculture; the figure is 36% in Germany (BMEL, 2020). Arable land can have a very different impact on floristic diversity, depending on the method of cultivation used (Hoffmann, 2012).

A considerable amount of food is produced by smallholder farms, some of which still use traditional farming methods on around 60% of the world's fields (Beck et al., 2016). In Central Europe, these methods were soon replaced by conventional farming, involving applications of chemical/synthetic pesticides (chemical plant protection products, or CPPP for short) and technical/technological developments from around 1950 onward and in some cases even earlier. Organic farming, a method of cultivation that does not use CPPP, became increasingly widespread in Central Europe from 1990 onward. In Germany, organic farming currently accounts for 10% of agricultural land (Ökolandbau.de, 2020). In most cases, farmers switched from conventional to organic farming. Traditional small‐scale farming where CPPP have never been used has almost completely disappeared from Central European farming areas. However, small pockets of arable land that have never been treated with CPPP still exist. In Germany, for example, such areas are referred in some cases to as arable weed reserves.

With the evolution of agriculture, fields have assumed important ecological functions for wild animals and plants, for example, for birds and flowering plant species (Ellenberg, 1988; Oppermann et al., 2012; Weijden et al., 2010). As such, about 10% of flowering plant species in Central Europe are dependent on field habitat (Meyer et al., 2013; Schneider et al., 1994) and are closely associated with this type of land. Floristic diversity differs relative to the method of cultivation. Organically farmed areas exhibit a significantly higher diversity of plant species than in conventionally cultivated areas, for example, Hole et al. (2005), Bengtsson et al. (2005), Glemnitz et al. (2006), Ponce et al. (2011), Batáry et al. (2013) and Tuck et al. (2014).

In the course of conventional cultivation, and as this type of farming intensified, considerable changes in the floristic composition of fields were determined (Albrecht et al., 2016; Hilbig & Bachthaler, 1992a, 1992b). These changes led to a decline in many locally adapted plant species, among other things. Meyer et al. (2013), for example, determined drastic declines in the species diversity and coverage of wild plants in arable fields as a result of long‐term intensification under conventional cultivation.

Comparative studies of conventional and organic farming came to the conclusion that both species diversity of plants in fields and the coverage of wild plants increased again significantly after land was switched to organic farming, for example, Hald (2008) and Stein‐Bachinger et al. (2020).

There was a striking correlation between the sharp decline in floristic diversity and the coverage of wild plants in fields and the comparably sharp decline in insect numbers detected in Central Europe (Hallmann et al., 2017, Seibold et al., 2019) and worldwide (Sanchez‐Byo & Wyckhuys, 2019). In spite of this correlation, the analyses were unable to adequately identify agricultural management as the cause of this decline. A large number of insects, such as wild bees and butterflies, depend on nectar and pollen from flowering plants in the landscape (Settele et al., 2015; Westrich, 2018). Since the large area of arable land influences the diversity and quantity of flowering plant species, and thus, their habitat functions for insects, the impact of cultivation methods on floristic diversity and on the flowering of plants in the landscape would need to be examined. Until now, however, no studies have recorded and analyzed the floristic diversity by species diversity and coverage in fields with addition of the actually flowering species and their flowering intensity relative to methods of cultivation in the agricultural landscape.

The aim of this study is therefore to determine the floristic diversity of spontaneously growing plant species in fields by species diversity, coverage, flowering species, and their flowering intensity. These characteristics are to be surveyed in parallel for conventional farming, organic farming, and smallholder farming where CPPP have never been used. This should facilitate an analysis of the effects of cultivation methods on floristic diversity, taking into account the flowering of plant species. The results should make it easier to assess the impact of cultivation methods on floristic diversity, including these systems' functions as sources of nectar and pollen for insects.

## 2 METHODS

### 2.1 Study area

The study area is located in Central Europe within the Federal State of Brandenburg, Germany, on the ‘Ostbrandenburger Platte’ natural unit, east of Berlin. The average annual temperature is 8.4°C, and average annual precipitation is 530 mm (Hoffmann et al., 2012). The region is an agricultural landscape typical of Central Europe, featuring a high proportion of agricultural land used primarily for food (cereals, oilseed rape) and feed production (alfalfa, maize). Around two thirds of the area, with sandy to slightly loamy diluvial soils, is used for agriculture. Arable land is increasingly being used for nonfood purposes, especially the cultivation of maize and cereals for bioenergy production. The intensity of land use has increased gradually in recent decades.

### 2.2 Investigation variants

The study focused on investigating three methods of cultivation: long‐term conventional arable farming, represented by (a) ‘conventional’; long‐term organic arable farming according to the rules of organic farming, without the use of CPPP, referred to as (b) ‘organic’; and smallholder farming where CPPP have never been applied, referred to as (c) ‘smallholder’.

In the case of (a), mineral fertilization and the annual multiple use of CPPP were common practice in recent decades (more than 50 years). Wild plants and spontaneously growing crops in fields were controlled by applying herbicides between one and three times each year.

(b) represents fields that have been farmed organically for a long time (29 years). The land was switched from conventional farming (more than 20 years before) to organic farming in 1991. CPPP have not been used there since then. Wild plants and spontaneously growing crops were regulated using mechanical methods and crop rotation, mainly by a crop rotation of cereals and alfalfa‒clover‒grass. CPPP have never been used in (c). Land use corresponds to traditional smallholder farming, which was common in the region until around 1950. Eight years ago, the fields were given the protection status of ‘arable weed reserve’ (Meyer & Leuschner, 2015) to ensure historical small‐scale farming in the long term. Since then, the land has been managed according to the criteria of organic farming.

### 2.3 Period of investigation

The field surveys, conducted in 2019, were carried out in two time windows, each lasting about 1 week from late March to early April (spring survey: spring) and late May to early June (early summer survey: summer). The two time windows were chosen to take into account the typical aspects of spring and early summer development of plants and their flowering under Central European conditions.

### 2.4 Vegetation surveys

The surveys were conducted on plots of 5 × 5 m each. To obtain representative data, in most cases, 12 plots per field were examined in spring and at the same place in summer. For every field, three plots each were surveyed at a distance of 2 m, 20 m, and 70 m to the field boundary, in addition to three plots in the middle of the field. All plots were located parallel to each other, with a distance of 30 m between each plot. The number of plots was based on the existing areas of the three cultivation methods (a), (b), and (c). The ratio of (a) to (b) to (c) in the study area was 3 to 1 to 0.1. As a result, 282, 48, and 26 plots were investigated in (a), (b), and (c), respectively, generating a total of 356 plots. The investigations were carried out in winter cereal crops that were typical for the regions (Table 1) and similar in the growth phenology.

**TABLE 1:** Cultivation variants (a) conventional, (b) organic, (c) smallholder, showing the arable crops (winter cereal) and number of plots (5 × 5 m) examined in spring and summer

Crops	(a)		(b)		(c)	
	Spring	Summer	Spring	Summer	Spring	Summer
Winter wheat	69	69	12	12	12	14
Winter barley	48	48	‐	‐	‐	‐
Winter rye	24	24	12	12	‐	‐
Spring/summer	141	141	24	24	12	14
Plots (a), (b), (c)	282		48		26	
Total of all plots	356					

The floristic species diversity of the spontaneously growing plant species, in short species diversity (SD) and the coverage of each of the plant species (C), was recorded for each plot based on an estimation scale by BRAUN‐BLANQUET (Braun‐Blanquet, 1964), which was extended (Table 2). To use the estimated values for calculations, numerical calculated values were defined for each category (see Table 2).

**TABLE 2:** Categories for coverage (r–5) with estimation scale (%) and assigned numerical calculated values (%) of plant species on the plots

Category	Estimation scale of coverage (%)	Numerical calculated values of coverage (%)
r	<<<1	0.01
r–+	<<1	0.05
+	<1	0.1
+–1	0.5	0.5
1a	1 to <3	2
1m	3	3
1b	>3 to <5	4
1–2	5	5
2	>5 to <25	15
2–3	25	25
3	>25 to <50	37.5
3–4	50	50
4	>50 to <75	62.5
4–5	75	75
5	>75 to 100	87.5

The flowering of species was determined simultaneously. This was done by visual estimation, dividing the flowering of species into six categories (Table 3). Records were made of which species in the plot had flowers (Categories 1–5) and which did not (Category 0). This enabled the number of flowering species (FS) per plot to be determined. Each species was assigned to one of the six categories of the estimation scale of flowering intensity (FI). C and FI are percentages calculated from the sum of individual values of species in a plot. The rule set out values ranging from 0% to 100%. If the numerical calculated values exceeded 100% coverage or flowering, the rule was deemed to be a maximum of 100%.

**TABLE 3:** Categories for flowering (0–5) of individuals of plant species on the plot with written explanation of flowering, the estimation scale of flowering intensity (%) and numerical calculated values of flowering intensity (%)

Category	Written explanation of flowering of individuals of a species	Estimation scale of flowering intensity (%)	Numerical calculated values of flowering intensity (%)
0	No flowers	0	0
1	A few single flowers	0.01 to 1	0.5
2	Low flowering	>1 to 5	3
3	Moderately numerous flowering	>5 to <25	15
4	Numerous flowering	>25 to 50	37.5
5	Very numerous flowering	>50 to 100	75

All field surveys were conducted by an accomplished botanist with many years of experience in assessing wild plants and crops on arable land.

### 2.5 Data analysis

The statistics software R (R Core Team, 2020) was used for statistical analysis. The differences in floristic diversity depending on the method of cultivation and the relationships between FS and FI to SD were investigated.

All numerical values from statistical analysis refer to the plots (*n* = 356) according to Table 1. SD is the number of species per plot; C is the sum of coverage of individual species per plot; FS is the number of flowering species per plot; and FI is the sum of the individual numerical calculated values of species' flowering intensity.

The spring and summer surveys were analyzed separately.

We analyze the effects of the method of cultivation on SD, C, FS, and FI by applying separate generalized linear model (GLM). The GLM for SD and FS (count data) applied to a quasipoisson distribution with ‘log‐link’ function, while for C and FI (percentages) applied to a quasibinomial distribution with ‘logit‐link’ function. Differences in the mean of SD, C, FS, and FI related to the method of cultivation were analyzed by chi‐square test.

Individual group differences for the cultivation methods—(a) and (b); (b) and (c); and (a) and (c)—for the four variables were examined using a post hoc test according to the Tukey Method.

Relationships between variable combinations FS and SD and between FI and SD were investigated by regression analysis using GLM. A GLM was based Gaussian distribution was applied to analyze FS as a dependent variable and SD as an explanatory variable. To analyze the effects of FS on FI, we applied a GLM based on quasibinomial distribution and ‘logit‐link’ function. ‘Explained deviation’ was calculated to assess the quality of the model.

Parameters SD, FS, C, and FI were aggregated to a numerical value for each variant to characterize the floristic diversity (FD) of cultivation methods (a), (b), and (c) and to compare them with each other. To this end, the medians m (mean of spring and summer medians) obtained in (c) for SD, FS, C, and FI were set equal to 100%. This results in percentage values for species diversity (SD), flowering species (FS), coverage (C), and flowering intensity (FI) from (c) to (a) according to Equations (1), (2), (3), (4):

(1) $${\text{SD}}_{\text{per}\;\text{cent}}=\frac{\left(\left({\text{Ma}}_{\text{SD}}\right)\times 100\right)}{\left({\text{Mc}}_{\text{SD}}\right)}$$

where Ma_SD_—median of cultivation variant (a) for SD; Mc_SD_ – median of cultivation variant (c) c_SD_;

(2) $${\text{FS}}_{\text{per}\;\text{cent}}=\frac{\left(\left({\text{Ma}}_{\text{FS}}\right)\times 100\right)}{\left({\text{Mc}}_{\text{FS}}\right)}$$

where Ma_FS_—median of cultivation variant (a) for FS; Mc_FS_—median of cultivation variant (c) c_FS_;

(3) $$C_{\text{per}\;\text{cent}}=\frac{\left(\left({\text{Ma}}_{C}\right)\times 100\right)}{{\text{Mc}}_{C}}$$

where Ma_c_—median of cultivation variant (a) for C; Mc_c_—median of cultivation variant (c) c_c_;

(4) $${\text{FI}}_{\text{per}\;\text{cent}}=\frac{\left(\left({\text{Ma}}_{\text{FI}}\right)\times 100\right)}{{\text{Mc}}_{\mathrm{FI}}}$$

where Ma_FI_—median of cultivation variant (a) for FI; Mc_FI_—median of cultivation variant (c) c_FI_.

In the same way, percentage values were calculated for species diversity (SD), flowering species (FS), coverage (C), and flowering intensity (FI) from (c) to (b).

These values were then calculated to determine a value of floristic diversity (FD) for each of the cultivation variants (a), (b), and (c) according to Equation 5:

(5) $$\text{FD}=\frac{\left({\text{SD}}_{\text{per}\;\text{cent}}+{\text{FS}}_{\text{per}\;\text{cent}}+C_{\text{per}\;\text{cent}}+{\text{FI}}_{\text{per}\;\text{cent}}\right)}{4}$$

## 3 RESULTS

**FIGURE 1:**
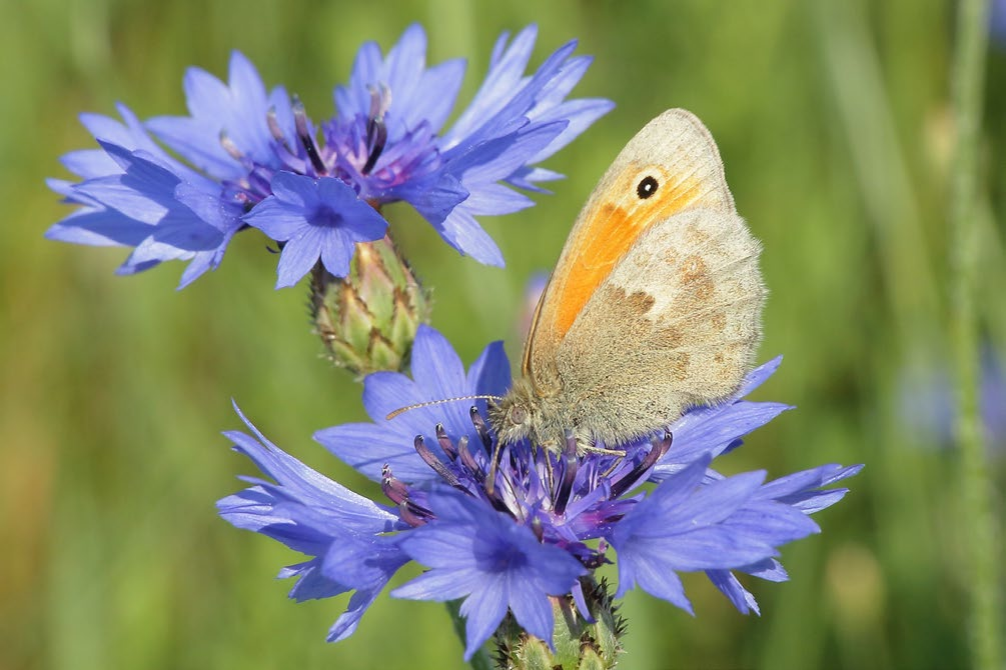
Flowers of *Centraurea cyanus* (the most common of the 106 wild plant species on the plots) as a nectar source for insects (on the example of the butterfly species *Coenonympha pamphilus*). Photograph: Jörg Hoffmann

A total of 106 spontaneously occurring plant species were detected in the 356 vegetation surveys of the plots. These included 95 wild plants (Figure 1) and 11 spontaneously growing crop species (*Brassica napus*, *Dactylis glomerata*, *Hordeum vulgare*, *Lolium multiflorum*, *Lolium perenne*, *Medicago sativa*, *Phacelia tanacetifolia*, *Secale cereale*, *Trifolium hybridum*, *Trifolium pratense*, and *Triticum aestivum*). The latter originated from arable crops cultivated in previous years. To be precise, species occurred 39 in (a) ‘conventional’ 63 in (b) ‘organic’ and 77 in (c) ‘smallholder’, respectively. Of these, 85 were herbs, 16 grasses, and 3 woody (Table 4).

**TABLE 4:** Botanical life forms (herbs, grasses, woody plants) of plant species on the plots on conventional (a), ecological (b) and small holder arable fields

Botanical life forms	(a)	(b)	(c)
Herbs	30	55	69
Grasses	8	7	7
Woody plants	1	1	1

### 3.1 Species diversity and method of cultivation in spring and summer

The methods of cultivation differed significantly in terms of SD when comparing groups (a), (b), and (c) in spring (*χ*² = 817.95; df = 2; *p* < .001) and summer (*χ*² = 855.57; df = 2; *p* < .001).

In individual comparisons, significant differences in SD were determined between (a) and (b) in spring and summer; between (a) and (c) in spring and summer; and between (b) and (c) in spring. The difference between (b) and (c) in summer constitutes an exception. In this case, there was a difference in tendency (Table 5).

**TABLE 5:** Ratio of mean (%) of methods of cultivation in terms of species diversity (SD), coverage (C), flowering species (FS), and flowering intensity (FI) in spring and summer: (a) conventional, (b) organic, (c) smallholder

Methods of cultivation	(a)/(b)		(a)/(c)		(b)/(c)	
	Ratio	*p*	Ratio	*p*	Ratio	*p*
SD—spring	0.112	<.0001	0.047	<.0001	0.416	<.0001
C—spring	0.045	<.0001	0.024	<.0001	0.536	.0613
FS—spring	0.000	<.0001	0.000	<.0001	0.250	<.0001
FI—spring	0.000	<.0001	0.000	<.0001	0.206	<.0001
SD—summer	0.166	<.0001	0.088	<.0001	0.533	<.0001
C—summer	0.049	<.0001	0.122	<.0001	2.496	.0316
FS—summer	0.069	<.0001	0.050	<.0001	0.728	.0024
FI—summer	0.018	<.0001	0.035	<.0001	1.932	.1052

**FIGURE 2:**
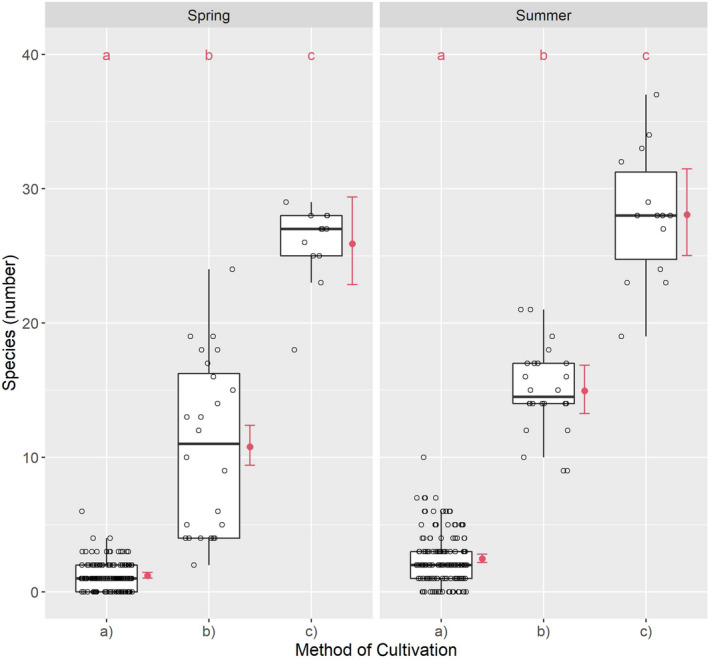
Species diversity relative to cultivation method: (a) conventional, (b) organic, and (c) smallholder. Different red letters indicate the significant differences by a Tukey test

Species diversity in spring was very low in (a); moderate to moderately high in (b); and high to very high in (c) (Figure 2) (Table 6). The median ratio was 1 to 11 to 27 from (a) to (b) to (c); it was 1 to 2.45 from (b) to (c).

**TABLE 6:** Median and mean ± standard error (SE) of species diversity (SD), coverage (C), flowering diversity (FS) und flowering intensity (FI) in the methods of cultivation (a) conventional, (b) organic, (c) smallholder in spring and summer

Methods of cultivation	(a)		(b)		(c)	
	Median	Mean ± SE	Median	Mean ± SE	Median	Mean ± SE
SD—Spring	1.00	1.21 ± 0.10	11.00	10.79 ± 0.76	27.00	25.92 ± 1.66
C—Spring	0.02	0.84 ± 0.24	7.38	15.89 ± 2.39	26.90	26.07 ± 4.06
FS—Spring	0.00	0.00 ± 0.00	1.50	2.33 ± 0.54	9.00	9.33 ± 1.55
FI—Spring	0.00	0.00 ± 0.00	0.75	5.04 ± 0.58	21.80	20.47 ± 1.52
SD—Summer	2.00	2.48 ± 0.15	14.50	14.96 ± 0.92	28.00	28.07 ± 1.64
C—Summer	0.02	3.49 ± 0.74	21.21	42.53 ± 4.83	24.05	22.87 ± 5.37
FS—Summer	1.00	0.67 ± 0.07	9.50	9.83 ± 0.62	12.00	13.50 ± 0.96
FI—Summer	0.50	0.95 ± 0.34	19.00	34.46 ± 4.03	17.00	21.39 ± 4.55

In summer, SD was slightly higher than in spring in all variants. It was low in (a); moderately high in (b); and high in (c) (see Table 6). In summer, the median ratio was 1 to 7.25 to 14.3 from (a) to (b) to (c) in summer; and 1 to 1.93 from (b) to (c).

### 3.2 Coverage and methods of cultivation in spring and summer

No individual coverage exceeded 100%. Coverage ranged from 0% to <100%.

**FIGURE 3:**
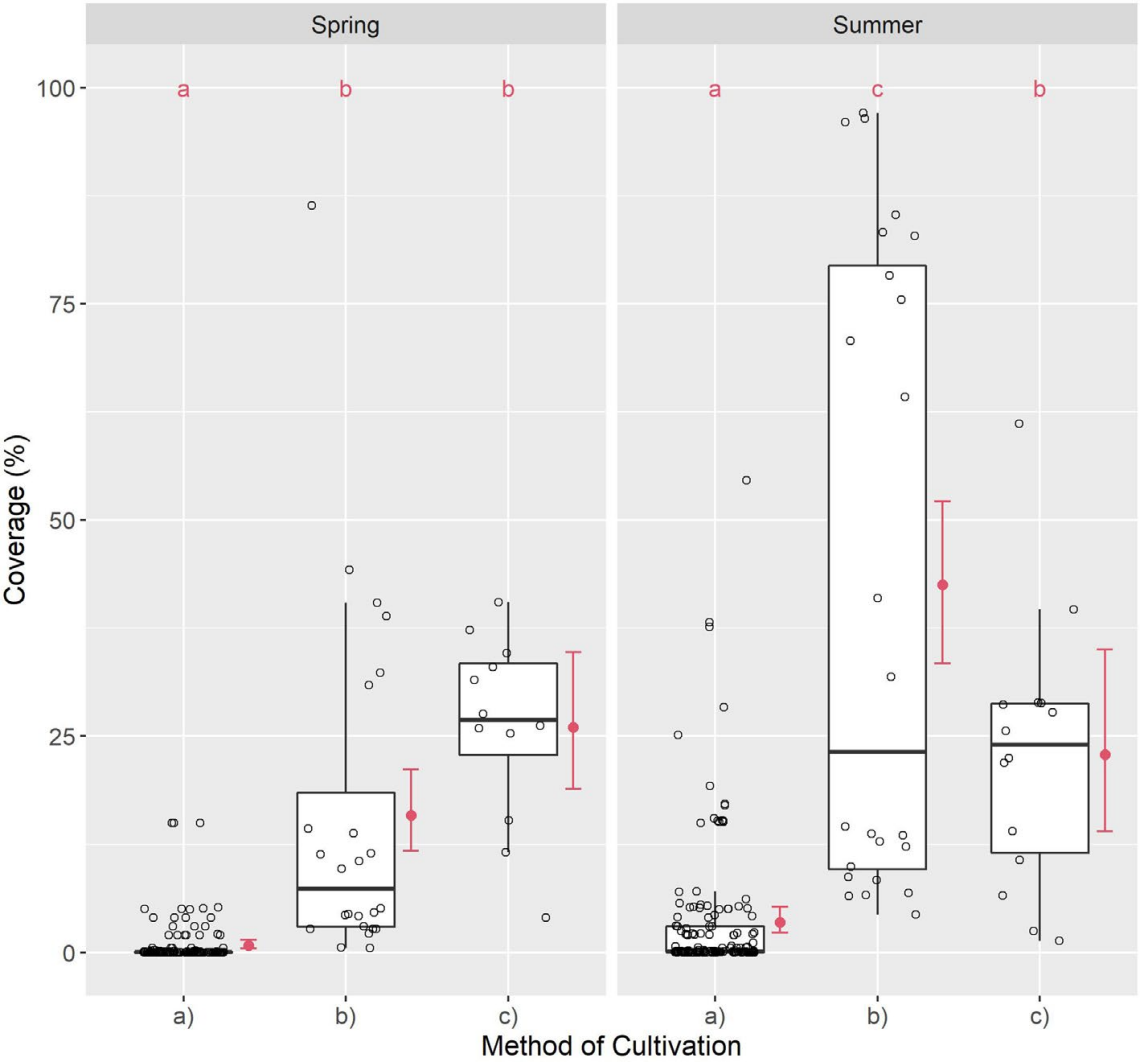
Coverage of plant species relative to cultivation method: (a) conventional, (b) organic, and (c) smallholder. Different red letters indicate the significant differences by a Tukey test

Coverage determined in the methods of cultivation differed significantly when comparing groups (a), (b), and (c) in spring (*χ*² = 112.39; df = 2; *p* < .001) and summer (*χ*² = 105.98; df = 2; *p* < .001). In the individual comparisons of cultivation methods, significant differences were found in (a) to (b) in spring and summer. In addition, (a) to (c) differed significantly in spring and summer, with *p* < .001, as did (b) to (c) in spring, with *p* = .0212. The difference between (b) and (c) in summer, with a broad scatter of individual values in (b), constitutes an exception (Figure 3).

In spring, (a) was almost free of spontaneous vegetation. C was very low in (a); low to moderate in (b); and high in (c) (see Table 6). The medium ratio was 1 to 369 to 1345 from (a) to (b) to (c); and 1 to 3.65 from (b) to (c) (see Table 5).

In summer, (a) was again almost vegetation‐free, in (b) C was sharp increasing; in (c) exhibited a slight increase. The median ratio was 1 to 1060 to 1202 from (a) to (b) to (c) and 1 to 1.13 from (b) to (c).

### 3.3 Flowering species and methods of cultivation in spring and summer

Flowering species differed significantly according to the method of cultivation when comparing groups (a), (b), and (c) in spring (*χ*² = 316.63; df = 2; *p* < .001) and summer (*χ*² = 664.35; df = 2; *p* < .001).

The differences in individual comparisons from (a) to (b) in spring and summer from (a) to (c) in spring and summer, and from (b) to (c) in spring were also significant. The difference between (b) and (c) in summer, with differences in tendency, constitutes an exception (Table 5).

**FIGURE 4:**
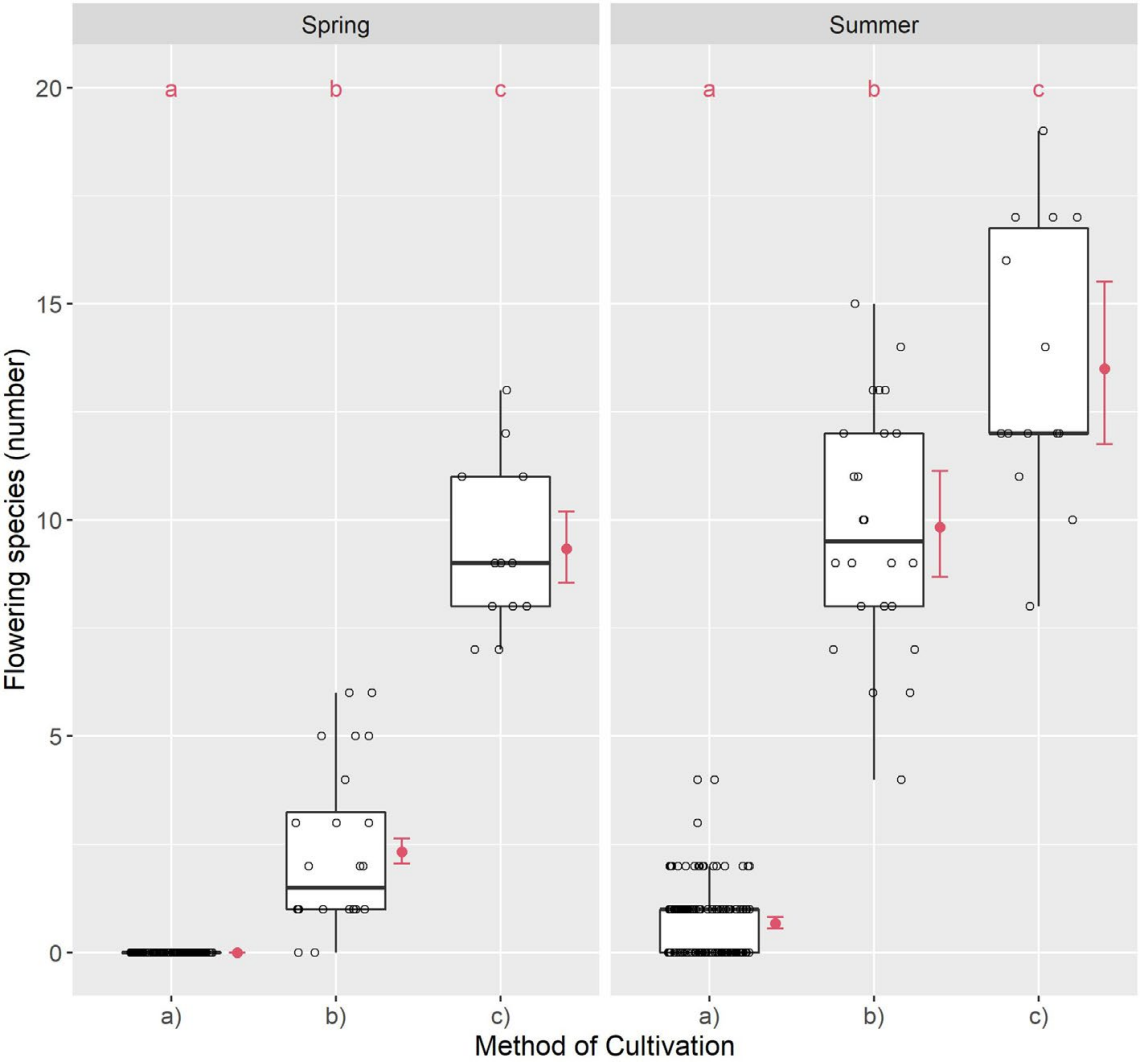
Flowering species relative to cultivation method: (a) conventional, (b) organic, and (c) smallholder. Different red letters indicate the significant differences by a Tukey test

The number of FS in spring was extremely low in (a); low in (b); and moderate in (c). In spring, the median ratio was 1 to 6 from (b) to (c). Relative to the median, no flowering occurred in (a), although FS were found in some plots (Figure 4). Due to the zero value of the median in (a), a numerical ratio from (c) to (b) and (a) was not relevant in spring. In summer, the number of FS was higher in all variants than in spring, albeit very low in (a); moderate in (b); and moderately high in (c) (Table 6). The median ratio was 1 to 9.5 to 12 from (a) to (b) to (c) in summer and 1 to 1.26 from (b) to (c).

### 3.4 Flowering intensity and methods of cultivation in spring and summer

No values of flowering intensity exceeded 100%. They ranged from 0% to <100%. Flowering intensity differed significantly according to the methods of cultivation when comparing groups (a), (b), and (c) in spring (*χ*² = 106.17; df = 2; *p* < .001) and summer (*χ*² = 130.62; df = 2; *p* < .001).

Differences in individual comparisons from (a) to (b) and from (a) to (c) in spring and summer were also significant. For (b) to (c), the difference was significant in spring, but not in summer (Table 5).

**FIGURE 5:**
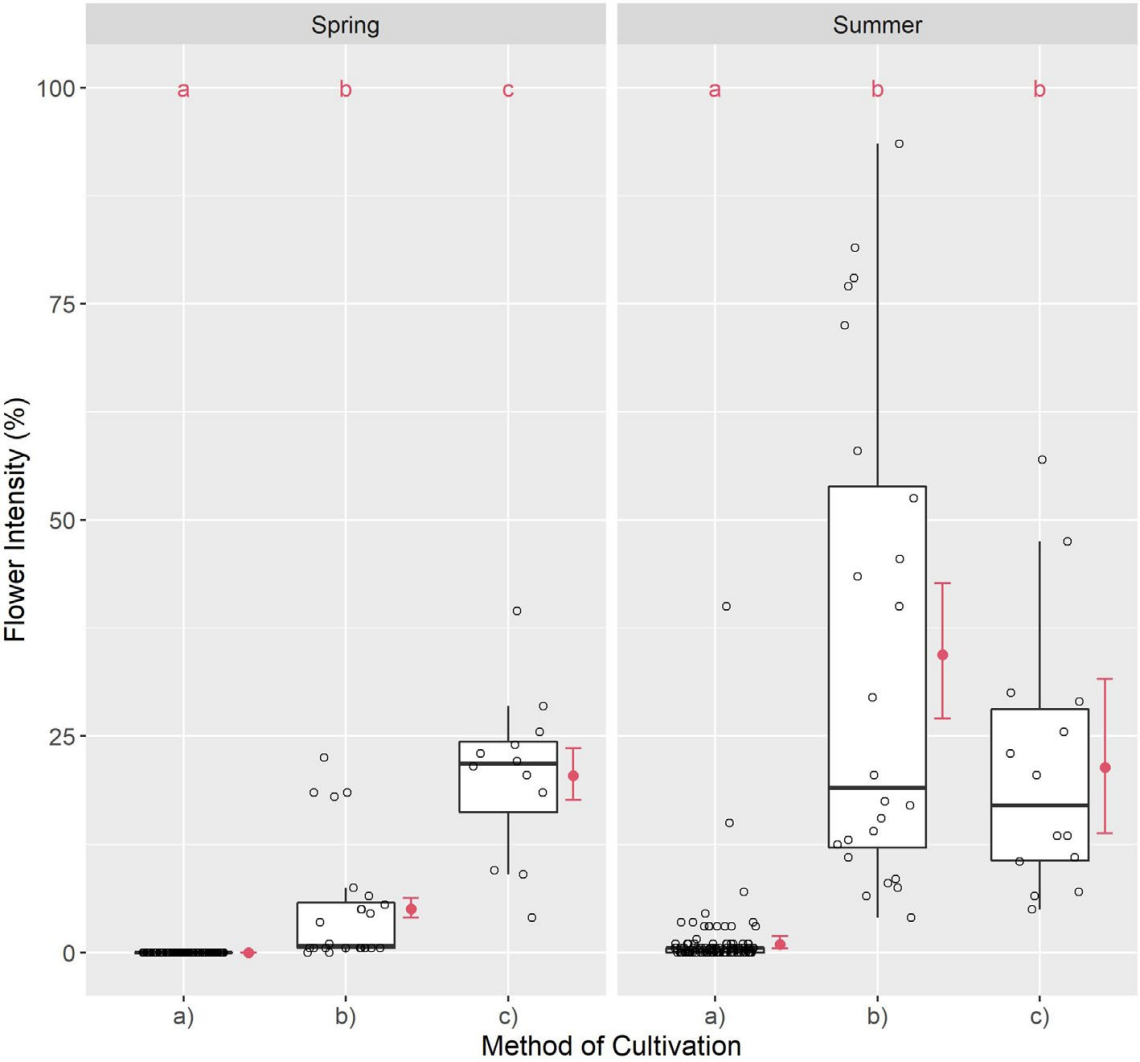
Flowering intensity relative to cultivation method: (a) conventional, (b) organic, and (c) smallholder. Different red letters indicate the significant differences by a Tukey test

In spring, no flowering intensity could be measured while (b) has low values and (c) has high values low in (b); but high in (c) (Figure 5) (Table 6). In spring, the ratio of (b) to (c) was 1 to 29.1 relative to the median and, FI was zero in (a), although individual plots also exhibited values above zero (cf. Figure 5). In summer, the median ratio was 1 to 38 to 34 from (a) to (b) to (c) and 1 to 0.89 from (b) to (c).

### 3.5 Relationships between species diversity and flowering species and between coverage and flowering intensity

**FIGURE 6:**
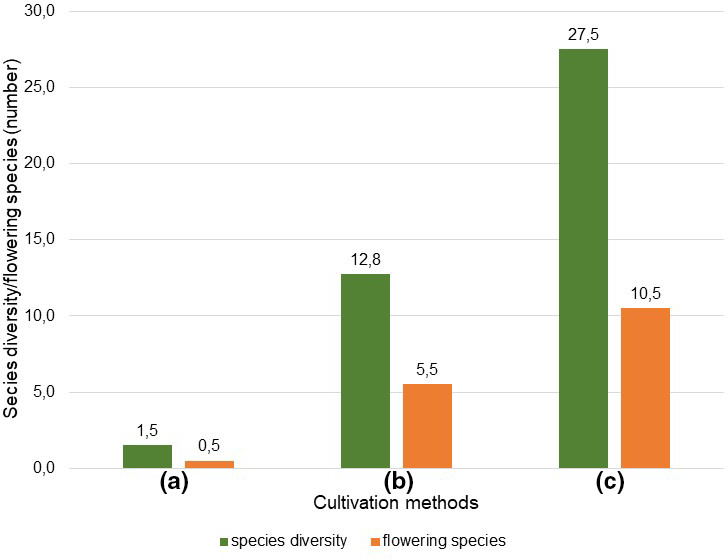
Median (spring and summer) of species diversity (SD) and flowering species (FS) of cultivation methods (c) smallholder, (b) organic, and (a) conventional

**FIGURE 7:**
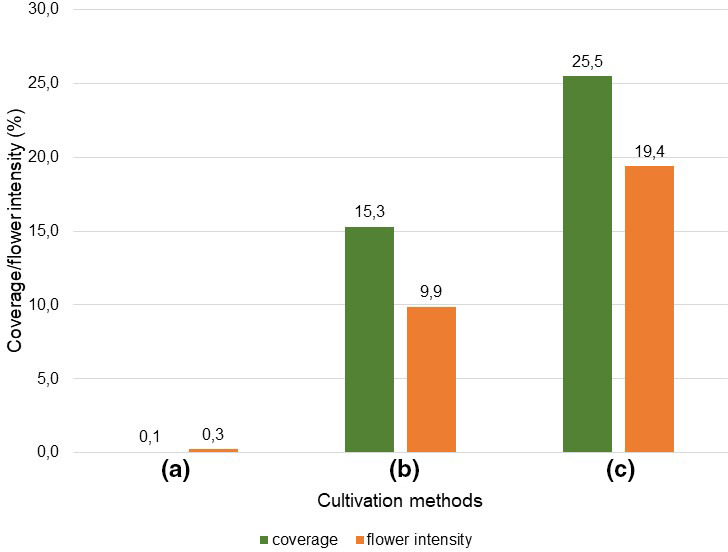
Median (spring and summer) of coverage (C) and flowering intensity (FI) of cultivation methods (c) smallholder, (b) organic, and (a) conventional

The relationships between species diversity and flowering species (Figure 6) and between coverage and flowering intensity are based on the median (Figure 7) (mean of spring and summer medians) (see Sections 3.1, 3.4) of cultivation methods (a), (b) and (c). The ratio of species diversity to flowering species was therefore 2.6 to 1 in (c), 2,3 to 1 in (b) and in (a) 3 to 1. The ratio of coverage and flowering intensity was 1.3 to 1 in (c) and 1.5 to 1 in b. In (c), both cover and flowering intensity were very low, and therefore, no ratio was determined.

### 3.6 Relationships between floristic diversity and methods of cultivation

**FIGURE 8:**
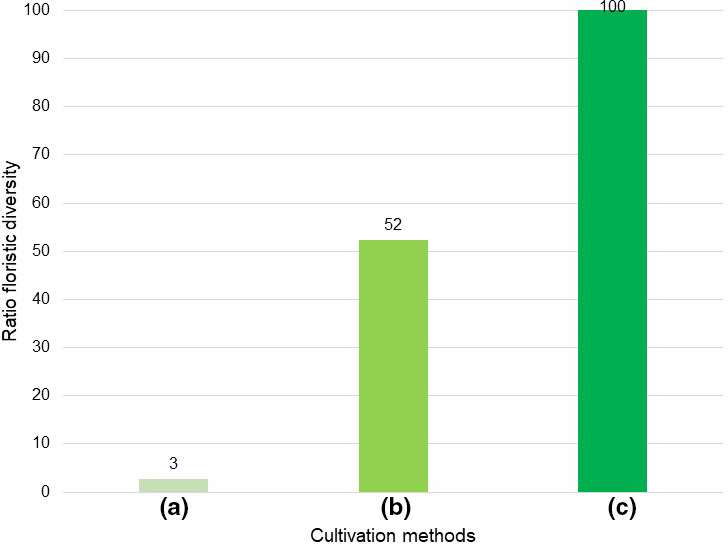
Relationships between floristic diversity in cultivation methods (c) smallholder to (b) organic and (a) conventional

Using the median of SD, FS, C, and FI for characterizing floristic diversity (FD) according to Eq. 5 resulted in a ratio of 100 to 52 to 3 from (c) to (b) to (a) (Figure 8).

Floristic diversity was in the ratio of 1 to 17.3 in (a) to (b); 1 to 1.91 in (b) to (c); and 1 to 33.3 in (a) to (c).

### 3.7 Flowering species and species diversity in spring and summer

**FIGURE 9:**
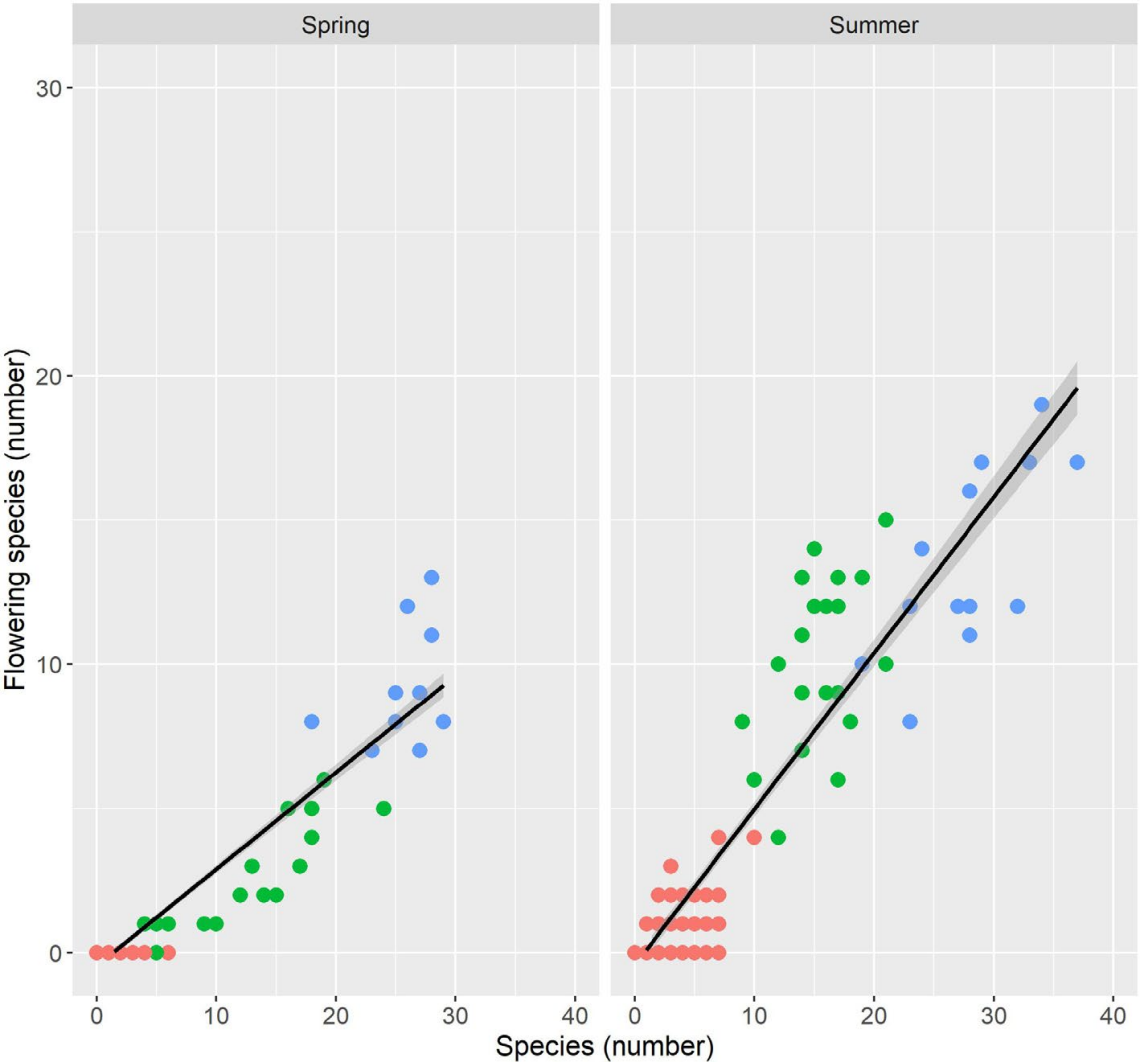
Relationship between species (number) and flowering species (number) in spring and summer: red dots (a) conventional; green dots: (b) organic; and blue dots: (c) smallholder

The relationship between FS and SD was significant in the sum of all cultivation methods (a), (b), (c) in spring (explained deviance = 0.907; *p* < .001) and summer (explained deviance = 0.882; *p* < .001). The increase in SD led to an increase in FS in spring, which follows the function *y* (spring) = 0.336*x* − 0.456. In summer, the share of FS in SD increased and followed the function *y* (summer) = 0.541*x* − 0.423. The number of FS increased more sharply relative to the increase in SD from spring to summer, characterized by a sharper increase in the linear function in summer compared to that in spring (Figure 9).

### 3.8 Flowering intensity and species diversity in spring and summer

**FIGURE 10:**
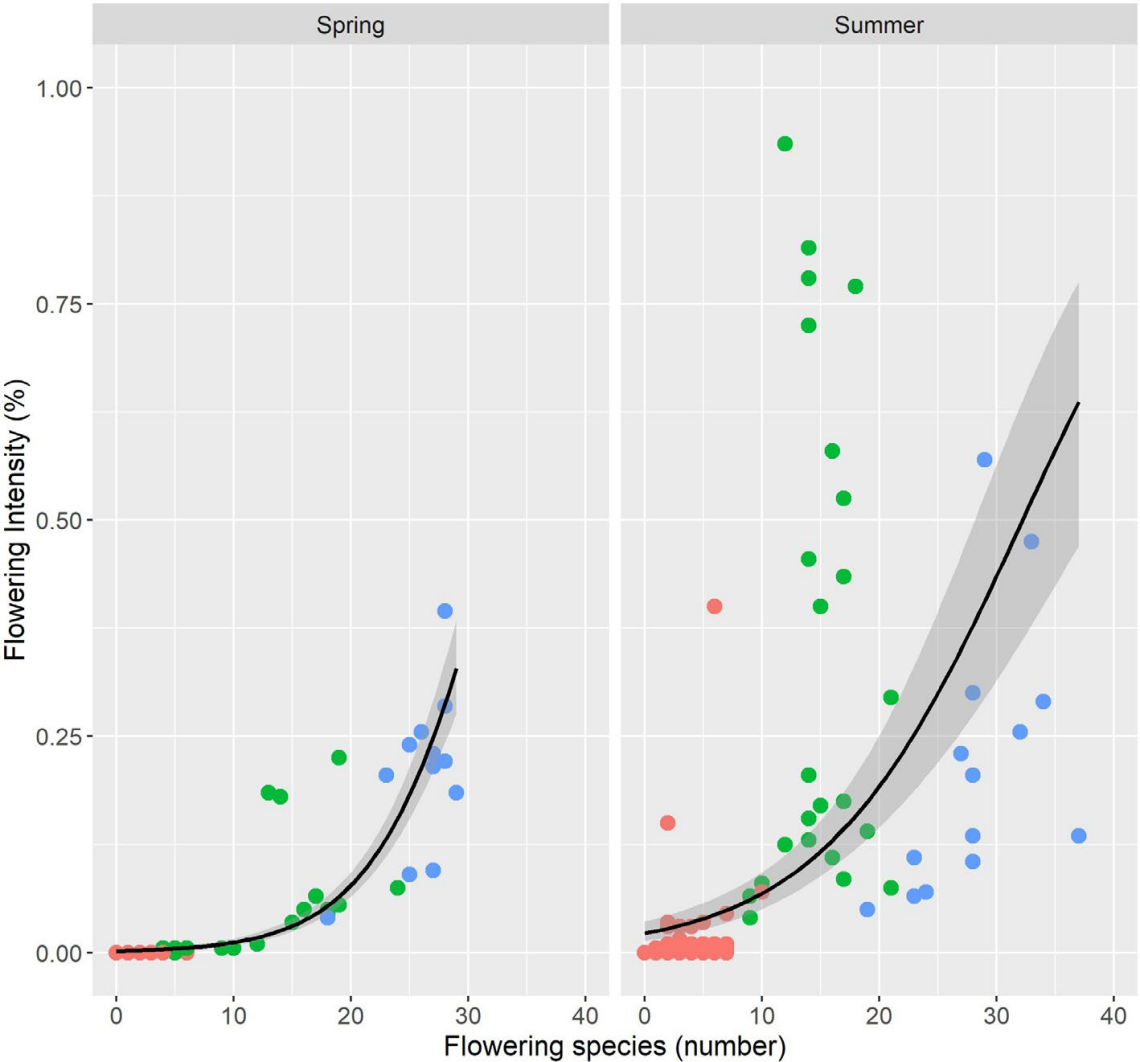
Relationship between flowering species (number) and flowering intensity (%) in spring and summer: red dots (a) conventional; green dots: (b) organic; and blue dots: (c) smallholder

The relationship between flowering intensity and species diversity (Figure 10) was significant in the sum of all cultivation methods (a), (b), (c) in spring (explained deviance = 0.825, *p* < .001) and summer (explained deviance = 0.333, *p* < .001).

In spring, the increase in species diversity led to an initially very small increase in flowering intensity (cultivation methods (a) and (b)), followed by a significant increase (cultivation method (c)), which follows the function $y\left(\text{spring}\right)=\frac{1}{\begin{array}{c} 1+\exp‐\left(0.195x‐6.377\right) \end{array}}$.

In summer, flowering intensity relative to species diversity increased compared to spring in all cultivation methods, but especially in (b), and followed the function: $y\left(\text{summer}\right)=\frac{1}{\begin{array}{c} 1+\exp‐\left(0.118x‐3.788\right) \end{array}}$.

## 4 DISCUSSION AND CONCLUSIONS

The differences determined in species diversity, coverage, actually flowering species and flowering intensity between cultivation methods (c) smallholder, (b) organic, and (a) conventional demonstrate a drastic decline in the floristic diversity of arable fields in the gradient from (c) to (a) caused by conventional methods of cultivation. This difference was greatest between smallholder farming, where CPPP had never been used, and conventional farming, where CPPP have been applied multiple times to arable fields annually for a long period.

A comparative analysis of the floristic diversity of (c), (b), and (a) on the basis of synchronously collected field data (as in this study) has so far been lacking in the literature. In contrast, there are comparative studies on species diversity and coverage for (b) and (a), for example, Hald (2008), Rahmann (2011), Batáry et al. (2013), and Stein‐Bachinger et al. (2020).

Findings on differences in cultivation methods between (b) and (a) in terms of species diversity and coverage of wild plants were reported by Ponce et al. (2011), for Mediterranean climate conditions in Southern Europe. In this case, species diversity and abundance of wild plant species were found to be 2.8 and 3 times higher, respectively, in organic farming than in conventional farming. However, no information was provided on the length of time that organic farming had been practiced. In our study, in which organic farming had been practiced for 29 years, species diversity relative to the median (spring, summer) was 8,56 times higher in (b) than in (a). Coverage in (b) was higher than in (a) by a factor of 153. This was because in (a), areas were almost free of spontaneous plant species in spring and summer, due to the use of herbicides.

Comparative studies have rarely reported, for example, Albrecht et al. (2020), on the method of cultivation used before switching to organic farming. For example, was arable land farmed conventionally for many years before switching to organic farming, and for how many years has organic farming been practiced?

If arable land is cultivated conventionally for a long period, for example, decades, before switching to organic farming, then a greatly reduced subset of spontaneously growing plant species can be assumed before the area was converted to (b). This would be reflected in all comparisons of (b) and (a). For Central European conditions, Hilbig and Bachthaler (1992a, 1992b), Kohlbecher et al. (2012) and Meyer et al. (2013) showed a decline in species diversity and coverage of wild plants in arable fields associated with long‐term conventional farming. This also points to initial conditions of floristic diversity when switching from (a) to (b).

The surveys conducted in (b) in our study took place almost three decades after these areas switched from conventional to organic farming in 1991. This means that floristic diversity was able to successively adapt to the specific cultivation system without chemical pesticides for 29 vegetation periods. The gradients of floristic diversity found in our study showed that there was significantly higher floristic diversity in (b) than in cultivation method (a). However, floristic diversity in (b) did not (yet) correspond to the much higher floristic diversity in (c), which represented smallholder farming where CPPP had never been applied.

In Central Europe, conventional methods of cultivation were introduced 60–70 years ago. In contrast, organic farming was usually only established after decades of conventional farming. It is therefore unlikely that floristic diversity will increase to a level of ‘previous’ floristic diversity just a few years after switching to organic farming. Lundkvist et al. (2008), for instance, found no increase in the number of wild plant species 15 years after arable areas were converted from conventional to organic farming.

Hilbig and Bachthaler (1992a, 1992b); Strokey et al. (2012); Richner et al. (2014) and Albrecht et al. (2016) pointed out that conventional farming led to substantial changes in the composition of wild plant species in arable fields, for example, rare and specialized species became locally extinct. Glemnitz et al. (2006) and Radics et al. (2004) also drew attention to this change in their studies conducted from Southern to Northern Europe. Several of these plant species are therefore unlikely to occur, even after a long period of organic farming.

Since intensive, conventional farming is practiced on more than 30% of Germany's land area, the results of this study indicate a landscape‐effective impoverishment of floristic diversity and, consequently, a very sharp decline in sources of nectar and pollen for insects.

The ratio of flowering species to species diversity for the methods of cultivation was about 1 to 3 in spring and about 1 to 2 (cf. functions Figure 9) in summer. Relative to the already very low level of species diversity in (a), these ratios indicate an even lower level of flowering species in (a). The number of flowering species increased from (a) to (b) to (c) in proportion to the increase in species diversity.

With regard to flowering species and flowering intensity in the methods of cultivation (cf. functions Figure 10), it was found that flowering intensity was generally also very low for (a), in line with the number of flowering species. When higher flowering intensity occurred in (a), it was only characterized by single dominant species such as *Centraurea cyanus* or *Papaver rhoeas*. In contrast, the much higher flowering intensity in (c) was characterized by a relatively large number of differently flowering species with higher diversity of nectar and pollen sources. In cultivation method (b), both dominant flowering species similar to (a) were found, as well as rarer species. These findings show that there are also major qualitative differences in flowering species between (c), (b), and (a). They demonstrate that the availability of different types of flowers in (c) also provides diverse sources of nectar and pollen for insects; this was less so in (b), and virtually non‐existent in (a).

If the dimension of the findings reflected the situation of floristic diversity and the supply of flowering plants in the agricultural landscape, this would have very great consequences for the functional biodiversity of fields as habitats for insects and other groups of species. If food resources for insects were characterized by species diversity, the number of individuals, flower availability, and the spatial connectivity of plant populations (in this case, coverage), then there would be an enormous deterioration of habitat conditions in (a), which partly explains the findings of a drastic decline in insects (Bennett et al., 2020; Hallmann et al., 2017). The degree of change in floristic diversity from (c) to (a) would also be an explanatory variable for biodiversity loss in agricultural landscapes with reference to other groups of species, for example, ground beetles (Heydemann & Meyer, 1983) and birds (Kamp et al., 2020; Langgemach et al., 2019). Biesmeijer et al. (2006) and Bennett et al. (2020) found parallel declines in pollinator insects (bee, noverfly) and insect‐pollinated plants.

The significant increase in organic farming in Germany over the past 30 years (Ökolandbau.de, 2020) has partly triggered a counter process by enhancing floristic diversity (Stein‐Bachinger et al., 2020). This was also detected in other regions, for example, by Norton et al. (2006), Wang et al. (2007); Wortman et al. (2010) and Rahmann (2011).

With regard to further studies, we recommend systematic investigations of the components of floristic diversity, including flowering species and flowering intensity, relative to existing cultivation systems or other types of habitat. The ‘relicts’ of smallholder farming in Central Europe where CPPP have never been used— almost lost type of farming that is very rare in this region—should be included in biodiversity monitoring. Among other things, this would generate a better understanding of the relationship between intensive farming and biodiversity and possibly improve biodiversity protection more efficiently by developing more sustainable cultivation systems.

## CONFLICT OF INTEREST

None declared.

## AUTHOR CONTRIBUTION

**Jörg Hoffmann:** Conceptualization (lead); Data curation (lead); Formal analysis (lead); Funding acquisition (lead); Investigation (lead); Methodology (lead); Project administration (lead); Resources (lead); Software (supporting); Supervision (equal); Validation (lead); Visualization (equal); Writing‐original draft (lead). **Tim Wahrenberg:** Conceptualization (supporting); Data curation (supporting); Formal analysis (supporting); Funding acquisition (supporting); Investigation (supporting); Methodology (supporting); Resources (supporting); Software (lead); Supervision (supporting); Validation (equal); Visualization (lead); Writing‐original draft (supporting); Writing‐review & editing (supporting).

## Data Availability

The data of all surveys are available in the database of the Federal Research Centre for Cultivated Plants (JKI), Institute for Strategies and Technology Assessment in Germany. The file is stored in DRYAD: https://doi.org/10.5061/dryad.vhhmgqnrv
